# Mailed participant reminders are associated with improved colonoscopy uptake after a positive FOBT result in Ontario’s ColonCancerCheck program

**DOI:** 10.1186/s13012-015-0226-0

**Published:** 2015-03-13

**Authors:** David Stock, Linda Rabeneck, Nancy N Baxter, Lawrence F Paszat, Rinku Sutradhar, Lingsong Yun, Jill Tinmouth

**Affiliations:** Sunnybrook Research Institute, Sunnybrook Health Sciences Centre, Toronto, Ontario Canada; Department of Medicine, University of Toronto, Toronto, Ontario Canada; Department of Radiation Oncology, University of Toronto, Toronto, Ontario Canada; Institute for Clinical Evaluative Sciences, Toronto, Ontario Canada; Dalla Lana School of Public Health, University of Toronto, Toronto, Ontario Canada; Institute of Health Policy Management and Evaluation, University of Toronto, Toronto, Ontario Canada; Cancer Care Ontario, Toronto, Ontario Canada; Department of General Surgery and Li Ka Shing Knowledge Institute, St. Michael’s Hospital, Toronto, Ontario Canada; Institute of Medical Sciences, University of Toronto, Toronto, Ontario Canada; Sunnybrook Health Sciences Centre, 2075 Bayview Ave Rm HG40, Toronto, ON M4N 3M5 Canada

**Keywords:** Fecal occult blood test, Colonoscopy, Colorectal cancer, Screening, Program evaluation

## Abstract

**Background:**

Timely follow-up of fecal occult blood screening with colonoscopy is essential for achieving colorectal cancer mortality reduction. This study evaluates the effectiveness of two ongoing interventions designed to improve colonoscopy uptake after a positive fecal occult blood test (FOBT) result within Ontario’s population-wide ColonCancerCheck program. The first was a revision of mailed FOBT lab results to physicians to explicitly define a positive FOBT and to recommend colonoscopy. The second was a letter to participants informing them of the positive FOBT and urging them to seek appropriate follow-up.

**Methods:**

Prospective cohort study using Ontario’s ColonCancerCheck program data sets (2008–2011), linked to provincial administrative health databases. Crude rate ratios were calculated to assess determinants of colonoscopy uptake among an Ontario-wide FOBT-positive cohort with rolling enrolment, followed from October 2008 through February 2011. Segmented time-series regression was used to assess the average additional change in colonoscopy uptake after FOBT-positive status following the introduction of two ongoing interventions among the same cohort.

**Results:**

A notification mailed directly to FOBT-positive screening participants was observed to increase colonoscopy uptake, beyond the modest average underlying increase throughout the study period, by an average of 3% per month (multivariable-adjusted RR: 1.03, 95% CI: 1.00–1.06). However, revision of the existing FOBT result notification to physicians was observed to have no effect.

**Conclusions:**

Direct participant notification of a positive FOBT result improved adherence with follow-up colonoscopy in Ontario’s population-wide ColonCancerCheck program. Further participant-directed interventions may be effective means of maximizing adherence in population-wide screening.

**Electronic supplementary material:**

The online version of this article (doi:10.1186/s13012-015-0226-0) contains supplementary material, which is available to authorized users.

## Introduction

In their lifetime, 1 in 13 men and 1 in 15 women are expected to develop colorectal cancer, the second and third most common cause of cancer mortality for Canadians, respectively [1]. Meta-analysis of four large randomized controlled trials (RCT) from Sweden, Denmark, England, and the United States has demonstrated biennial fecal blood screening capable of reducing colorectal cancer mortality by approximately 15% [2,3]. When adjusted for mean screening attendance, this risk was reduced by 25% (relative risk: 0.75; 95% CI: 0.66–0.84) [3]. However, diagnostic colonoscopy after a positive fecal occult blood test (FOBT) is essential for FOBT-derived colorectal cancer mortality reduction.

Canadian provinces have begun to implement population-wide fecal occult blood screening programs, the first being Ontario’s ongoing ColonCancerCheck (CCC), implemented in April 2008 [4] by Cancer Care Ontario (CCO). The CCC Program provides biennial screening with guaiac FOBT kits (Hemoccult-II), distributed primarily by primary care physicians (PCP), for average-risk individuals between the ages of 50 and 74 years, inclusive, and recommends follow-up of a positive result with colonoscopy. In Ontario, colonoscopies are provisioned at the discretion of the PCP or specialist, who has been instructed to do so following a positive FOBT result. Over 3.9 million Ontario residents are estimated to be within this age range [5], comprising a substantial average-risk target screening population. As of 2011, 74.6% of Ontarians receiving a positive FOBT result proceeded to follow up colonoscopy within 6 months [4]. While up from 63.4% in 2008, this fell short of early results from European population-wide screening programs implemented around the same time [6,7] and targets based on Canadian consensus [8] guidelines. Factors most strongly associated with failure to follow up with colonoscopy in Ontario are having had recent colonoscopy and ordering of repeat FOBT [9].

To increase colonoscopy uptake following a positive FOBT, CCC introduced two ongoing strategies as of February and October of 2010. The first was a revision of the existing FOBT result notification to PCPs to include an explicit definition of a positive FOBT result and recommend timely follow-up with colonoscopy. The second was a mailed letter to participants, informing them of their positive FOBT result, and recommending them to seek appropriate follow-up with their PCP. This study aimed to assess the effect of these two strategies on colonoscopy uptake.

## Methods

### Study population

Our population-wide perspective cohort comprised all screening-aged Ontarians (50 to 74 years) who received a positive FOBT result from September 1, 2008, through February 28, 2011, in the CCC program. In Ontario’s CCC, FOBT participants collect two samples each from three consecutive spontaneously passed stools onto individual FOBT card windows. One or more positive samples, out of six windows, define a positive FOBT result.

FOBT-positive participants were eligible if they were alive at the index date, defined henceforth as the date of their first positive FOBT result within the study period, following sample return. Exclusion criteria consisted of a previous diagnosis of colorectal cancer (ICD-9: 153.0 to 153.4 and 153.6 to 154.1, inclusive); a positive result from an FOBT ordered by a registered nurse, pharmacist, or via Telehealth Ontario (rather than by a PCP); or missing information on age, sex, or postal code. Ethics approval was granted by the Sunnybrook Research Ethics Board.

### Data sources

The FOBT-positive cohort was identified using the CCC Laboratory Reporting Tool (LRT) administrative database. The LRT tracks the distribution of CCC-provided FOBT kits and records results. The Ontario Health Insurance Plan (OHIP), Ontario Cancer Registry (OCR), Colorectal Interim Reporting Tool (CIRT), Canadian Institute for Health Information (CIHI), and the Registered Persons Database (RPDB) provided data required for baseline exclusions, the ascertainment of outcome status, and the derivation of covariates. The CIRT captures the occurrence, findings, and quality-related measures of colonoscopies performed in participating hospitals. Colonoscopies performed outside of participating hospitals are captured by OHIP. The RPDB contains demographic information such as age, sex, and postal code from all those registered for OHIP coverage. Detailed descriptions of OHIP, OCR, CIHI, and RPDB have been published elsewhere [10,11]. Access to, and linking of, the above databases was facilitated by the Institute for Evaluative Clinical Sciences (ICES), authorized by comprehensive data-sharing agreements with Ontario’s Ministry of Health and Long Term Care and CCO. ICES maintains anonymized administrative data on health care utilization on all OHIP-insured Ontarians.

### Strategies to improve colonoscopy uptake

In Ontario, the PCP who orders the FOBT is responsible for organizing follow-up after a positive test. To improve the uptake of colonoscopy after a positive FOBT result, CCC implemented two ongoing interventions, beginning February and October of 2010. The first (strategy 1) was a revision of the mailed, standardized notification to the ordering PCP to explicitly define a positive FOBT result as one indicating at least one of six positive test kit windows. PCPs were reminded of the corresponding recommended course of action as follows: “Timely follow up with colonoscopy is strongly recommended to rule out colorectal cancer.” The second (strategy 2) was a letter (Additional file 1) mailed directly to participants informing them of a positive FOBT result and urging them to contact a PCP or nurse practitioner to discuss appropriate diagnostic follow-up. Prior to the introduction of this intervention, participants did not receive notification directly from the CCC program of their positive FOBT result. Strategies 1 and 2 were implemented as of February and October, 2010, respectively. Both were coexistent following the onset of strategy 2.

### Study variables

#### Outcome

As there is no way to reliably identify screening colonoscopy within OHIP records over the duration of the study period, the outcome was colonoscopy irrespective of indication. Colonoscopy dates were identified using CIRT and OHIP databases. For the latter, events were identified by the billing code Z555 with any of the following subcodes: E740, E741, E747, or E705, indicating colonoscopies complete to splenic flexure, hepatic flexure, cecum, and terminal ileum, respectively. The earliest colonoscopy service date following a positive FOBT result in either CIRT or OHIP databases was used to indicate timing of the outcome. In Ontario, OHIP is a single payor for the majority of health services, including colonoscopy, resulting in strong incentivization for submission of OHIP billing claims. As such, we expect the vast majority of colonoscopy outcomes to have been captured.

#### Covariates

Covariates included age, sex, socioeconomic status (SES), health region, aggregate comorbidity, continuity of primary care, repeat FOBT within 6 months post index positive FOBT, and occurrence of recent colonoscopy within 5 years prior to the index date. Health region was defined by Local Health Integration Network (LHIN), ranked by 2007 fiscal year colonoscopy rates, in descending order of colonoscopies per capita. SES status was an aggregate measure, derived from average household income by census dissemination area, divided into quintiles. Due to the substantial variability of income in rural dissemination areas, those living in such regions were assigned a sixth category. A comorbidity index was derived using Aggregated Diagnosis Groups™ (ADG®) of the Johns Hopkins Adjusted Clinical Group (ACG®) case-mix system, previously validated for use with Ontario administrative health data [12]. Continuity of care was captured using the Usual Provider Continuity (UPC) density measure, a commonly used index for this construct [13]. UPC is the ratio of the number of visits to a usual primary care provider to all other primary care providers. A ratio of 0.74 or lower was considered indicative of low continuity of care [14]. For this study, the usual care provider was constrained as the PCP who ordered the index positive FOBT.

### Statistical analysis

Segmented linear [15] Poisson regression was used to assess the effect of the two ongoing interventions (strategies 1 and 2) on colonoscopy uptake among FOBT-positive CCC participants. The outcome was colonoscopy rate per month. The numerator for this rate was the number of colonoscopies per month and the denominator was person-time contributed by FOBT-positive individuals each month. This model provided estimation of the baseline rate, the linear trend in rate (i.e., slope) prior to strategy 1, the difference in linear trend in rate pre- and post-strategy 1, and the difference in linear trend in rate pre- and post-strategy 1 and 2 combined. Crude and multivariable-adjusted rate ratios (RR) representing the estimated relative monthly average change in colonoscopy rate, and additional change post-strategy 1 and post-strategy 1 and 2, are presented with 95% confidence intervals. Analyses were conducted using SAS 9.3 (SAS Institute, Cary, North Carolina).

Person-time contribution was truncated at date of death and follow-up was constrained to a maximum of 3 months: an individual that had a positive FOBT in 1 month remained eligible for the outcome during the following 2 months. This restriction facilitated the assessment of strategy 2, for which only 5 months of follow-up were available (October 2010 to February 2011, inclusive) due to the introduction of a competing intervention. Additionally, it focused results on colonoscopy uptake within a timeframe approximately consistent with the 2-month wait time recommended by the Canadian Association of Gastroenterology [8], a target adopted by the CCC program [16]. To verify whether modeled follow-up length may have substantially altered estimated intervention effects, we performed a sensitivity analysis excluding the strategy 2 study interval, allowing FOBT-positive participants to remain at risk for colonoscopy for up to 6 months.

## Results

There were 81,283 person-months contributed by 39,105 Ontarians between 50 and 74 years of age with a positive FOBT result between October 2008 and February 2011, inclusive. The proportion of person-time contribution prior to strategy 1, between strategy 1 and 2, and post-strategy 2 was 50.2, 27.7, and 22.1 persons-months, respectively, while 46.3%, 28.0%, and 25.7% of colonoscopy service dates fell within these respective intervals. There were 13,229 colonoscopies following a positive FOBT result during the 29-month study period.

Person-time contribution and observed colonoscopy rates by positive FOBT participant characteristics, aggregated over the entire follow-up, are summarized in Table 1. The colonoscopy rate was 6% higher for those aged 50 to 69 (crude RR: 1.06; 95% CI:1.01–1.11), compared to those 70–74 years, at index-positive FOBT. It was 10% higher in the highest, relative to all other, urban SES quintiles (crude RR: 1.10, 95% CI: 1.05–1.15), while rural participants exhibited a 16% lower rate compared to those in urban areas (crude RR 0.84; 95% CI: 0.79–0.89). It was substantially lower for participants who completed a repeat FOBT within 180 days following the index-positive FOBT (crude RR: 0.24; 95% CI: 0.21–0.27) or for those that had a colonoscopy within the past 5 years (crude RR: 0.67; 95% CI: 0.63–0.70).

**Table 1:** **Person-time contribution and observed colonoscopy rates across index-positive FOBT participant characteristics from Ontario’s ColonCancerCheck program, October 2008 through February 2011**

**Characteristics**	**Person-months (%)**		**Rate** ^**a**^
	**Total = 81,283**		
Age at index FOBT+ (years)			
50 to 59	36,381	(44.8)	16.4
60 to 69	32,235	(39.7)	16.4
70 to 74	12,667	(15.6)	15.5
Sex			
Females	36,600	(45.0)	16.2
Males	44,684	(55.0)	16.3
Urban SES quintiles/rural status			
(lowest) Urban quintile 1	13,308	(16.4)	15.4
Urban quintile 2	15,320	(18.8)	16.4
Urban quintile 3	14,898	(18.3)	17.2
Urban quintile 4	14,805	(18.2)	16.7
(highest) Urban quintile 5	13,315	(16.4)	17.1
Rural	9,631	(11.8)	13.9
LHIN^b^			
(Highest) LHIN 1	3,644	(4.5)	11.7
LHIN 2	6,444	(7.9)	13.5
LHIN 3	4,749	(5.8)	16.6
LHIN 4	9,034	(11.1)	12.5
LHIN 5	9,150	(11.3)	16.9
LHIN 6	1,297	(1.6)	12.4
LHIN 7	4,010	(4.9)	13.3
LHIN 8	4,086	(5.0)	17.4
LHIN 9	3,481	(4.3)	11.1
LHIN 10	5,861	(7.2)	16.4
LHIN 11	6,211	(7.6)	17.1
LHIN 12	2,022	(2.5)	20.0
LHIN 13	10,385	(12.8)	18.1
(Lowest) LHIN 14	10,919	(13.4)	21.6
ADG score^c^			
0 or 1	9,169	(11.3)	16.3
2 or 3	25,476	(31.3)	16.5
4 or 5	21,637	(26.6)	16.8
6 or 7	13,438	(16.5)	16.4
≥8	11,559	(14.2)	14.7
Usual provider continuity index^d^			
Low	30,399	(37.4)	16.0
High	50,885	(62.6)	16.5
Repeat FOBT^e^			
Yes	7,421	(9.1)	4.2
No	73,860	(90.9)	17.5
Prior colonoscopy			
None	67,328	(82.8)	17.2
0 to 2 years	5,283	(6.5)	8.1
2 to 5 years	8,921	(11.0)	13.4

^a^Observed rate per 100 person-months.

^b^Health region ranked by colonoscopy rates for the 2007 fiscal year.

^c^Derived Advanced Diagnosis Group index.

^d^High indicates that 75% or more of primary care services in the 2 years prior to the index-positive FOBT were performed by the same physician.

^e^Within 6 months post index FOBT.

**Figure 1:**
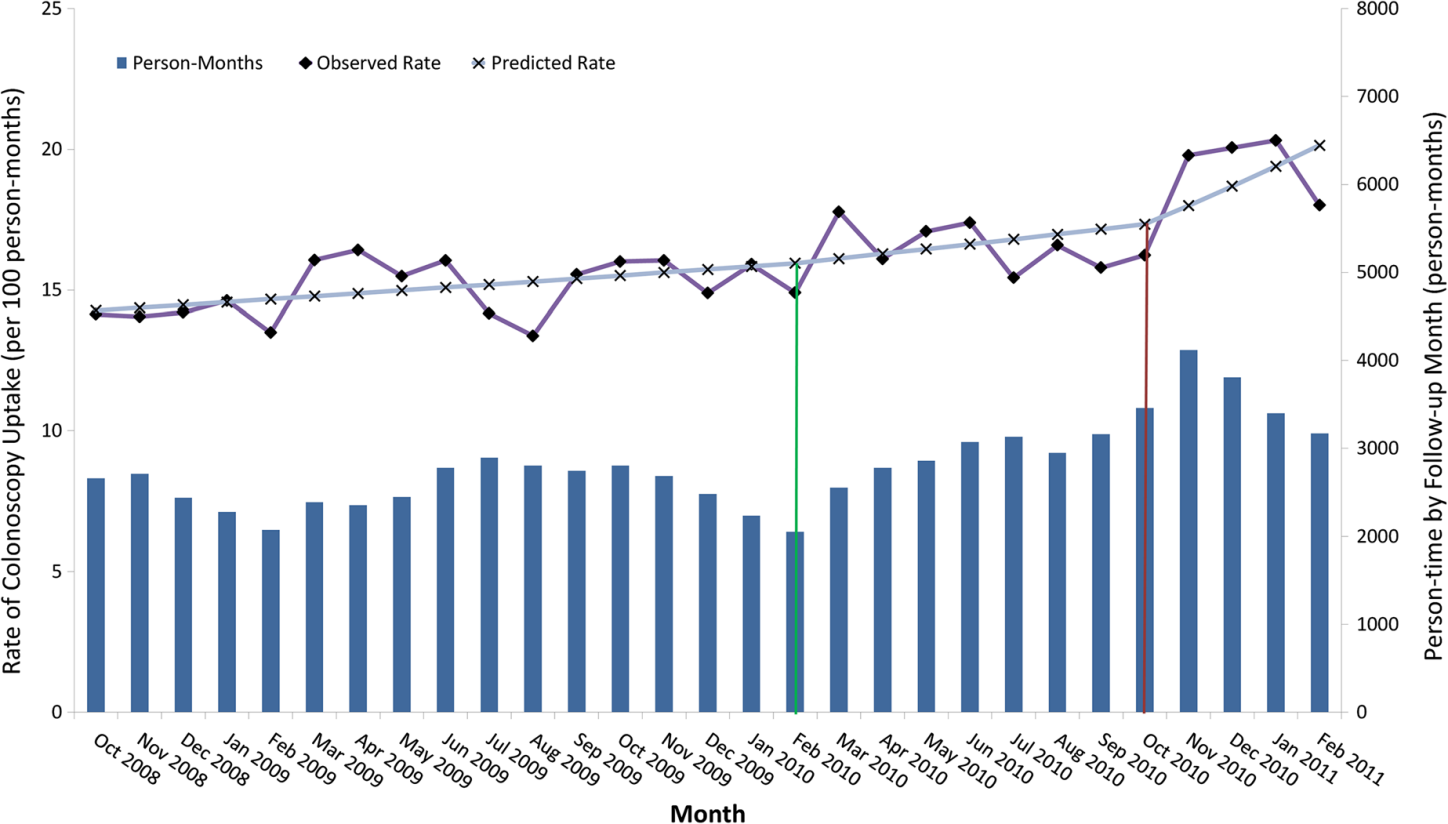
**Observed and predicted rates of colonoscopy uptake by follow-up month from Ontario’s** ***ColonCancerCheck*** **program.** Green and brown vertical lines indicate implementation of strategies 1 and 2, respectively.

Model-estimated colonoscopy rates increased by 7.8%, 8.7%, and 16.1% prior to implementation of strategy 1, between implementation of strategy 1 and 2, and post-implementation of strategy 2, respectively (Figure 1). Rate ratios estimating the crude and multivariable-adjusted average monthly change in colonoscopy uptake are presented in Table 2. Prior to strategy 1, there was a borderline statistically significant 1% per month increase in colonoscopy utilization (multivariable-adjusted RR: 1.01; 95% CI: 1.00–1.01). After the implementation of strategy 1, and prior to strategy 2, there was no additional increase in estimated colonoscopy uptake. For post-strategy 2, there was a borderline statistically significant 3% (multivariable-adjusted RR: 1.03; 95% CI: 1.00–1.06) additional average increase, per month, in colonoscopy uptake. Under the modeled constraint that the average background increase in colonoscopy utilization was constant over the entire 29 months of follow-up, strategy 2 led to an approximate 16% relative increase in colonoscopy uptake between October 2010 and February 2011, inclusive. Due to concerns that a maximum follow-up time of 3 months may not provide enough time for the interventions to exert an effect on colonoscopy uptake, a model allowing a 6-month maximum cohort membership was explored for strategy 1. Results were not materially different to those summarized in Table 1 (data not shown).

**Table 2:** **Crude and multivariable-adjusted rate ratios indicating average monthly change in colonoscopy uptake from October 2008 through February 2011 in Ontario’s** ***ColonCancerCheck*** **program**

	**Rate ratio**	**95% CI**	***p*** **value**
Pre strategy 1			
Crude	1.01	(1.00–1.01)	0.002
Multivariable-adjusted^a^	1.01	(1.00–1.01)	0.002
Strategy 1^b^			
Crude	1.00	(0.99–1.02)	NSS^d^
Multivariable-adjusted^a^	1.00	(0.99–1.01)	NSS^d^
Strategy 2^c^			
Crude	1.03	(1.00–1.05)	0.04
Multivariable-adjusted^a^	1.03	(1.00–1.06)	0.04

^a^Adjusted for age, sex, SES, LHIN, total ADG score, usual provider continuity index, repeat FOBT after index positive FOBT, and prior colonoscopy. Each covariate was entered in the multivariable Poisson model as indicated in Table 1.

^b^Revision of standardized reporting of positive FOBT result to PCPs. Implemented February 1, 2010.

^c^Reporting of positive FOBT result directly to participants. Implemented October 1, 2010.

^d^Not statistically significant.

## Discussion

To our knowledge, this is the first population-wide study to directly evaluate interventions implemented to improve colonoscopy uptake among Ontario’s CCC program participants following a positive FOBT result. Strategy 2, mailed notifications to participants including recommended course of action, was observed to increase colonoscopy uptake, beyond the modest average underlying increase throughout the study period. Strategy 1, mailed notifications to PCPs to specify that colonoscopy is recommended, was observed to have no effect. Crude-rate ratios corroborate other findings from Ontario [9] indicating that the strongest determinants of not proceeding to colonoscopy are repeat FOBT and recent colonoscopy, followed by factors related to socioeconomic status.

The literature evaluating interventions specifically designed to improve colonoscopy uptake after a positive FOBT result is sparse. The existing evidence demonstrates efficacy of those that target physicians and efficiency of health care infrastructure. A 48% increased odds of complete diagnostic evaluation (OR: 1.48; 95% CI: 1.04–2.11) was observed in a US study assessing the combined impact of physician reminders and practice-tailored educational outreach [17]. The study included 2,992 FOBT-positive patients over the age of 50 from 318 participating primary care practices. A major discrepancy between this physician reminder and notifications utilized in Ontario’s CCC program was the emphasis on feedback in the former: participating physicians in the US study were asked to indicate whether diagnostic follow-up had been recommended and performed and provide examination dates and diagnoses reached. PCP correspondence in the Ontario CCC program does not solicit feedback, perhaps increasing the likelihood of them going unheeded. However, the tracking of PCP decision making in population-wide programs as large as Ontario’s CCC would require extensive upgrades to centralized health information infrastructure beyond what is currently implemented in most settings, Ontario included.

The majority of published intervention studies have compared positive FOBT follow-up adherence pre- and post-electronic medical record system and infrastructure augmentations. Three studies conducted in US Veteran Affairs (VA) centers found compelling evidence that automatic gastroenterology referrals [18] and electronic chart reminders with prompting of physician feedback verifying appropriate action had been taken [19], or manual-tracking resulting in further reminders in cases of undocumented recommended follow-up [20], improve adherence. A fourth VA-center-based prospective cohort study with historical controls found that automatic gastroenterology referrals reduced time to (*p* < 0.0001), though not proportion of (*p* = 0.77) patients completing colonoscopy [21]. A fifth study within a large Seattle-based integrated healthcare organization reported substantial increases in recommended follow-up after the adoption of electronic record tracking and, subsequently, a manual audit system, propelling complete diagnostic evaluation rates markedly higher than the national average [22]. In contrast, FOBT results and subsequent reminders in Ontario’s CCC are not maintained in a centralized electronic medical record database accessible to the PCP, are not accompanied by automatic colonoscopy referrals, and do not prompt PCP feedback verifying further action has been taken. It should be emphasized that the scope of Ontario’s CCC greatly exceeds that of the settings in which the above interventions were evaluated. If improved communication with PCPs can contribute to achieving colonoscopy uptake targets, those that require extensive individualized tracking may be not be feasible in large population-based programs such as Ontario’s CCC.

The literature specifically assessing strategies that directly target participants is limited. A smaller Korean trial found that telephone reminders following mailed notifications resulted in a higher proportion of colonoscopy acceptance after a positive FOBT than mailed notifications alone (*p* = 0.038) [23]. The feasibility of telephone contact with each FOBT-positive participant in Ontario’s CCC is uncertain. Additionally, telephone correspondence may be no more effective at improving compliance with follow-up colonoscopy. A recent Italian RCT found mailed invitations to be equally as effective as those conveyed by telephone on this outcome [24].

Though personalized mailed notifications appear to increase colonoscopy uptake in population-wide screening, it is uncertain whether these alone will be sufficient in elevating colonoscopy uptake to match other programs. France’s population-based screening program, including 46 out of 96 departments and the same age range as Ontario’s CCC, reported that 88% of those receiving a positive FOBT throughout 2008 and 2009 proceeded to follow up colonoscopy, 90% of which occurred within 5 months [7]. England’s Bowel Cancer Screening Programme, initiated in July of 2006, indicated that 83% of the first million screened with FOBT in the program proceeded to colonoscopy [6]. While this proportion reflects only those invited to participate in the first round of screening (not a true population-wide sample), the elevated adherence could be due to automatic referrals. In the United Kingdom, follow-up colonoscopy appointments are centrally scheduled upon a positive FOBT result, while in Ontario, organizing follow-up is the domain of the PCP. The implementation of automatic referrals may be dependent on the integration of electronic medical record systems beyond those that currently exist in Ontario, the adoption of which trails that of the UK and other European countries [25].

### Strengths and limitations

These findings should be interpreted in the context of the following strengths and limitations. To our knowledge, this is the first population-based study assessing interventions specifically designed to improve colonoscopy uptake rates among FOBT-positive participants in an organized screening program. Furthermore, it is the first to evaluate the impact of interventions on this outcome in a post-pilot phase population-wide setting. Access to such a large, representative FOBT-positive cohort produces results that are arguably more generalizable to the growing number of such programs worldwide [26].

A limitation is the 3-month maximum follow-up, making performance comparisons with other population-wide screening programs that have reported higher rates of follow-up colonoscopy after longer intervals [6,7] of uncertain utility. A second related limitation is the relatively short study interval after implementation of strategy 2, allowing for the modeling of only five time points. As such, inferences made about strategy 2 are based on fewer data and thereby may be more susceptible to bias such as those stemming from variation in colonoscopy utilization due to seasonal trends. We acknowledge that this observational design does not support a causal link as strongly as an RCT allowing randomized allocation of interventions and contemporaneous comparison to controls. However, as implementation of strategies 1 and 2 was province-wide we did not have access to a suitable control group. Validation of the effectiveness of direct participant notification of screening outcomes in improving follow-up colonoscopy compliance is pending future work.

## Conclusions

Our results suggest that further refinement of mailed correspondence notifying PCPs of FOBT results and reminding them of the recommended follow-up protocol will be of little benefit in increasing colonoscopy uptake among FOBT-positive individuals. In contrast, our findings indicate that increased participant engagement may help large population-wide programs improve adherence. Thus, it is recommended that such programs consider interventions that target the participant directly. Based on experience in other jurisdictions, additional potentially useful strategies for population-wide screening programs such as Ontario’s CCC include system-level changes such as the implementation of centralized automatic colonoscopy referral for persons with positive FOBT results.

## Additional file

Additional file 1:
**Sample letter to CCC participants reporting a positive FOBT result.** The letter is mailed directly to participants informing them of a positive FOBT result and urging them to contact a PCP to discuss appropriate diagnostic follow-up.

## Abbreviations

CCC: ColonCancerCheck
CIHI: Canadian Institute for Health Information (database)
CIRT: Colonoscopy Interim Reporting Tool (database)
FOBT: Fecal occult blood test
ICES: Institute for Clinical Evaluative Sciences
LHIN: Local Health Integration Network
LRT: Laboratory Reporting Tool (database)
OCR: Ontario Cancer Registry (database)
OHIP: Ontario Health Insurance Plan (database)
PCP: Primary care physician
RCT: Randomized controlled trial
RPDB: Registered Persons Database (database)
UPC: Usual Provider Continuity
