# Supplementary material for: Mailed participant reminders are associated with improved colonoscopy uptake after a positive FOBT result in Ontario’s ColonCancerCheck program
Source: Implement Sci. 2015 Mar 13;10:35. doi: 10.1186/s13012-015-0226-0 (PMC4371630; doi:10.1186/s13012-015-0226-0)
Supplement: Additional file 1: — Sample letter to CCC participants reporting a positive FOBT result. The letter is mailed directly to participants informing them of a positive FOBT result and urging them to contact a PCP to discuss appropriate diagnostic follow-up. [file 13012_2015_226_MOESM1_ESM.pdf]

APPENDIX 1. Sample Letter to Patients Reporting Positive FOBT Result

ColonCancerCheck | ContrôleCancerColorectal

<Correspondence Template>

<Letter Reference Number>

<Person First Name> <Person Last Name>
<Address Line 1>
<Address Line 2>
<City> <Province> <Postal Code>

Dear <Person First Name> <Person Last Name>:

We are writing to let you know about your Fecal Occult Blood Test (FOBT) result.

Your test result shows that some blood was found in your stool sample. This is an ‘abnormal’ test result. There could be a number of reasons why blood was found. Most people who have blood found in their stool do **not** have colorectal cancer. However, your result does mean that **you will need a procedure to find the cause of the bleeding.**

It is important to talk to a family physician or nurse practitioner about your result and the procedure that is needed to find out why you have blood in your stool. A copy of your result was sent to your family physician or nurse practitioner. If you have not heard from their office, please contact them within the next two weeks to discuss your result.

If you have any questions about this letter please call us toll free at **1-866-662-9233** from Monday to Friday, 8:30 a.m. to 5:00 p.m. Or, visit www.Ontario.ca/ColonCancerCheck.

Yours sincerely,

Linda Rabeneck

Linda Rabeneck, MD, MPH, FRCPC
Vice President, Prevention and Cancer Control
Cancer Care Ontario

It is important to Cancer Care Ontario (CCO) that all Ontarians with disabilities can access the services and information we provide. To receive this information in another format, contact CCO Public Affairs:

☎ (416) 217-1821 TTY (416) 217-1815 publicaffairs@cancercare.on.ca

Ontario
